# C-Reactive Protein Inhibits Survivin Expression via Akt/mTOR Pathway Downregulation by PTEN Expression in Cardiac Myocytes

**DOI:** 10.1371/journal.pone.0098113

**Published:** 2014-05-27

**Authors:** Beom Seob Lee, Soo Hyuk Kim, Jaewon Oh, Taewon Jin, Eun Young Choi, Sungha Park, Sang-Hak Lee, Ji Hyung Chung, Seok-Min Kang

**Affiliations:** 1 Graduate Program in Science for Aging, Yonsei University, Seoul, Republic of Korea; 2 Cardiology Division, Severance Cardiovascular Hospital and Cardiovascular Research Institute, Yonsei University College of Medicine, Seoul, Republic of Korea; 3 Severance Integrative Research Institute for Cerebral and Cardiovascular Diseases (SIRIC), Yonsei University Health System, Seoul, Republic of Korea; 4 Department of Applied Bioscience, College of Life Science, CHA University, Gyeonggi-do, Republic of Korea; 5 Brain Korea 21 Project for Medical Science, Yonsei University College of Medicine, Seoul, Republic of Korea; University of South Alabama, United States of America

## Abstract

C-reactive protein (CRP) is one of the most important biomarkers for arteriosclerosis and cardiovascular disease. Recent studies have shown that CRP affects cell cycle and inflammatory process in cardiac myocytes. Survivin is also involved in cardiac myocytes replication and apoptosis. Reduction of survivin expression is associated with less favorable cardiac remodeling in animal models. However, the effect of CRP on survivin expression and its cellular mechanism has not yet been studied. We demonstrated that treatment of CRP resulted in a significant decrease of survivin protein expression in a concentration-dependent manner in cardiac myocytes. The upstream signaling proteins of survivin, such as Akt, mTOR and p70S6K, were also downregulated by CRP treatment. In addition, CRP increased the protein and mRNA levels of PTEN. The siRNA transfection or specific inhibitor treatment for PTEN restored the CRP-induced downregulation of Akt/mTOR/p70S6K pathway and survivin protein expression. Moreover, pretreatment with a specific p53 inhibitor decreased the CRP-induced PTEN expression. ERK-specific inhibitor also blocked the p53 phosphorylation and PTEN expression induced by CRP. Our study provides a novel insight into CRP-induced downregulation of survivin protein expression in cardiac myocytes through mechanisms that involved in downregulation of Akt/mTOR/p70S6K pathway by expression of PTEN.

## Introduction

C-reactive protein (CRP), which is an acute-phase protein, has been described as a non-specific biomarker of inflammation and risk factor for cardiovascular disease (CVD) [1]. CRP plays a crucial role in the expression of adhesion molecules of endothelial cells, the progression of atherosclerotic lesion, survival of endothelial progenitor cell, activation of monocytes and expression of tissue factor, the key initiator for thrombosis [2]–[5]. Recently, Nagai et al. documented that CRP enhanced pressure overload-induced cardiac remodeling through inflammatory response [6]. Our previous study showed that CRP induces p53-mediated cell cycle arrest in H9c2 cardiac myocytes [7].

Survivin is a unique member of the inhibitor of apoptosis gene family and its expression is an important factor in regulating proper cell division and apoptosis [8]–[10]. It is also known that the cellular stress response to an anti-apoptotic and mitotic checkpoint is maintained by survivin [11]. It has been demonstrated that phosphotidyliositol-3-kinase (PI3K), Akt and p70S6K1 pathway is essential for regulating survivin expression in human ovarian cells and prostatic cancer cells [12], [13]. And, survivin plays a role in the insulin-induced anti-apoptotic effect in the ischemic-reperfused heart through PI3K/Akt/mammalian target of rapamycin (mTOR) signaling pathway [14]. It was also reported that ventricular function was decreased in a survivin knock-out mouse model [15]. Furthermore, reduction of survivin expression is associated with induced apoptosis and pressure-overload cardiac remodeling process in spontaneously hypertensive rat [16]. Recently, we demonstrated that anti-apoptotic effect of survivin in doxorubicin-induced cell death in H9c2 cardiac myocytes [17]. Therefore, we can speculate the possible interaction between CRP and survivin in the process of cell survival pathway. To our knowledge, the effect of CRP on survivin expression in cardiac myocytes has not been determined. In the present study, we investigated whether and how CRP would regulate survivin expression in cardiac myocytes.

## Methods and Materials

### Reagents and antibodies

Human CRP protein was purchased from Millipore. To remove sodium azide from the commercial CRP preparation, CRP was repeatedly filtered with Tris buffer (10 mM Tris, 100 mM NaCl and 2 mM Ca^2+^) until remaining 0.0001% sodium azide using Ultrafilter Vivaspin 500 (Sartorius). Anti-survivin, anti-phospho-p53 (Ser15), anti-p53, anti-phospho-Akt (Ser473), anti-Akt, anti-phospho-mTOR (Ser2481) and anti-mTOR antibodies were obtained from Cell Signaling. Anti-PTEN, anti-phospho-p70S6K (Thr421/Ser424), anti-p70s6k, anti-phospho-ERK1/2 (Tyr204), anti-ERK1/2 and anti-GAPDH antibodies were purchased from Santa Cruz Biotechnology. BpV (PTEN inhibitor), U0126 (ERK inhibitor), SP600125 (JNK inhibitor), CGK733 (ATM/ATR inhibitor) and NU7026 (DNA-PK inhibitor) were purchased from Calbiochem. PFT-α (p53 inhibitor) was obtained from Sigma-Aldrich.

### Cell culture

The rat heart-derived myoblast cell line, H9c2 cardiac myocytes, was obtained from the American Type Culture Collection. H9c2 cardiac myocytes were maintained in Dulbecco’s modified Eagle’s medium (DMEM) supplement with 10% fetal bovine serum (FBS), 100 U/ml of penicillin and 100 µg/ml of streptomycin (Gibco) at 37°C in a humidified atmosphere with 5% CO_2_. All experiments were performed using cells between 15 to 25 passage numbers. H9c2 cardiac myocytes were incubated for 24 hours in 100 mm culture plate and changed to 0.5% FBS for 24 hours starvation. After starvation, cells were pretreated with 0 ∼ 50 µg/ml CRP in 0.5% FBS for 24 hours.

Neonatal rat cardiac myocytes were also isolated from the Sprague-Dawley rats on postnatal day 1 to 2. Cells were preplated (2 hours) to enrich for cardiac myocytes, plated at a density 1500 cell/mm^2^, and cultured for 48 hours in Minimum Essential Medium (MEM)-α containing 10% FBS, 100 U/ml of penicillin, 100 µg/ml of streptomycin (Gibco) and 100 µmol/L boromodeoxyuridine (Sigma). At 48 hours after plating, the culture media was replaced with 0.5% FBS MEM-α. As assessed by immunofluorescence with an antibody against α-sarcomeric actin and α-smooth muscle actin, >95% of the isolated cells were cardiac myocytes (data not shown).

### Immunoblot analysis

Cells were solubilized in a cell lysis buffer (Cell Signaling) and centrifuged at 14,000 rpm for 1 hour at 4°C. The protein samples were separated by a SDS-polyacrylamide gel and then electrotransferred to polyvinylidene difluoride membranes. The membranes were washed with Tris-buffered saline-tween 20 (TBS-T) and blocked by incubation with 10% nonfat dry milk in TBS-T for 1 hour at room temperature. The membranes were incubated with indicated primary antibodies for overnight. After washing, they were incubated with horseradish peroxidase-conjugated secondary antibody for 1 hour and subjected to enhanced chemiluminescence detection. The loading control was performed on the same membrane after stripping.

### Reverse transcription-polymerase chain reaction (RT-PCR)

Total ribonucleic acid (RNA) isolated from cardiac myocytes using QIAzol-Regent (Qiagen) was reverse transcribed using Omniscript Reverse Transcriptase (Qiagen). The cDNAs was amplified using *Taq* deoxyribonucleic acid (DNA) polymerase (Takara, Japan). The following primer sequences were used: survivin primers 5'-ATGGGTGCTACGGCGCTGCCC-3' and 5'-TCAGCGTAAGGCAGCCAGCTG-3', PTEN primers 5'-AGACCATAACCCACCACAGC-3' and 5'-TTACACCAGTCCGTCCTTTCC-3', GAPDH primers 5'-AATGCATCCTGCACCACCAACTGC-3' and 5'-GGAGGCCATGTAGGCCATGAGGTC-3'. Polymerase chain reaction (PCR) products were separated by electrophoresis in a 1% agarose gel containing Gel-red (Biotium).

### siRNA transfection

Scrambled control siRNA were purchased from Santa Cruz Biotechnology. PTEN siRNA targeting sequences, 5'-CAAGAUCUUCACAAAAGGGUU-3', was obtained from Genolution Pharmaceuticals. Cells were transfected with 20 nM of siRNA using Lipofectamine RNA iMAX (Invitrogen) according to the manufacturer’s protocol.

### Immunofluorescence microscopy

Cells incubated on Lab-Tek chamber slides (Nalgene Nunc). The cells were fixed with 3% paraformaldehyde for 10 min at room temperature and washed with PBS. Cells were permeabilized in 0.5% Triton X-100 buffer (0.5% Triton X-100, 20 mM Hepes-KOH, pH 7.9, 50 mM NaCl, 3 mM MgCl_2_, 300 mM sucrose) in PBS for 10 min and washed with PBS. They were blocked with PBS containing 0.3% goat serum and 5% bovine serum albumin for 1 hour at room temperature and then incubated for 1 hour with α-sarcomeric actin antibody (dilution 1∶200; Thermo) and α-smooth muscle actin antibody (dilution 1∶200; Abcam). Cells were washed with PBS and incubated with Alexa fluor 488 and rhodamine Red-X (dilution 1∶500; Invitrogen) as secondary antibody for 1 hour in dark room. After washing, the cells were mounted with ProLongantifade reagent containing DAPI. The immunoreactive signals were visualized by confocal laser scanning microscope LSM700 (Carl Zeiss, Germany).

### Statistical analysis

All measured data was displayed as average ± S.E. The differences between the groups were compared by the unpaired one-way analysis of variance and Student’s *t*-test, followed by post *hoc* analysis Bonferroni test. Differences were considered significant at *P*<0.05.

## Results

### CRP inhibits survivin expression in H9c2 cardiac myocytes

**Figure 1:**
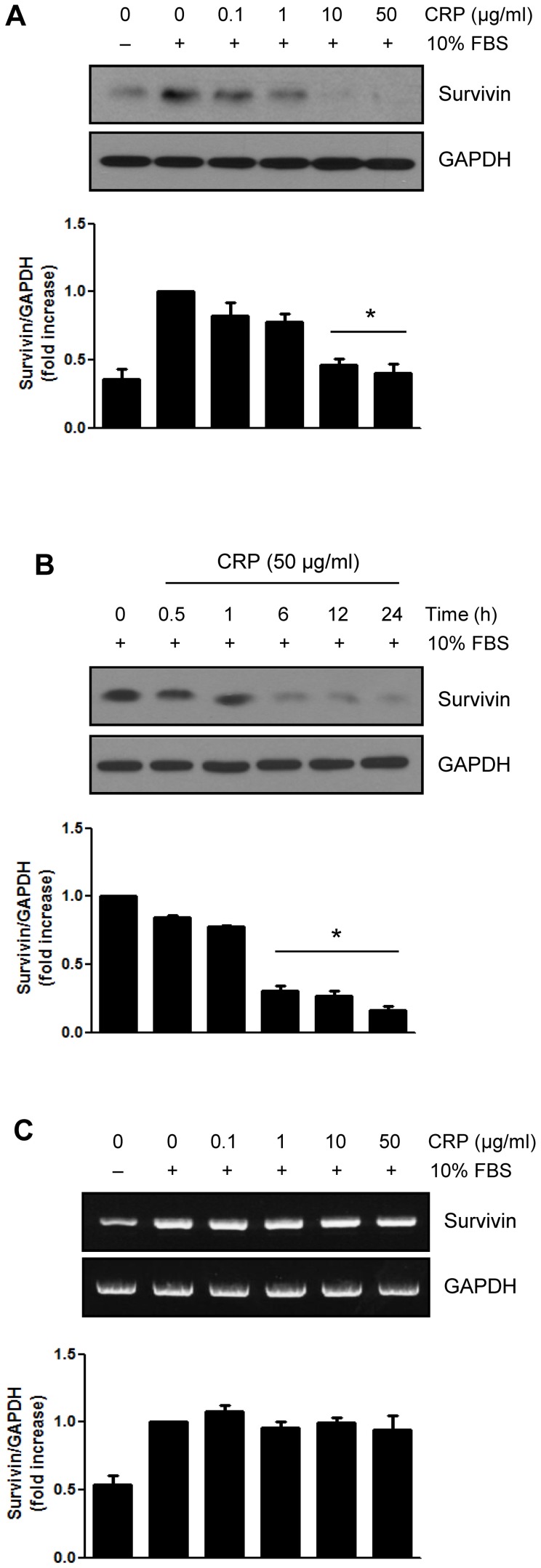
Effect of CRP on protein and mRNA level of survivin in H9c2 cardiac myocytes. (A) H9c2 cardiac myocytes were pretreated for 24 hours with various concentrations of CRP in 0.5% FBS and then treated 10% FBS for 24 hours. Whole cell lysates were Immunoblot analysis with anti-survivin antibody. *P<0.05 compared to control (10% FBS treatment without CRP). (B) H9c2 cells were pretreated with 50 µg/ml of CRP for indicated times and then treated 10% FBS for 24 hours. Protein levels of survivin were analyzed by immunoblot assay. *P<0.05 compared to control. (C) H9c2 cells were pretreated for 24 hours with CRP in 0.5% FBS and then treated 10% FBS for 24 hours. Total RNA was purified and subjected to RT-PCR. All experiments were performed independently at least three times. Data are mean ± S.E.

We first investigated the effect of CRP on survivin expression in H9c2 cardiac myocytes. Cells were pretreated with various concentrations of CRP with 0.5% FBS for 24 hours and incubated with 10% FBS for 24 hours. As shown in Fig. 1A, serum-deprived H9c2 cardiac myocytes exhibited low expression of baseline endogenous survivin. After 24 hours incubation with 10% FBS, survivin expression level was significantly increased. However, survivin protein levels were decreased by CRP in a concentration-dependent manner (Fig. 1A). Fig. 1B shows the time-dependent decrease of survivin protein level, when pretreated with 50 µg/ml of CRP for variable time. We further studied changes in mRNA level of survivin by CRP pretreatment. RT-PCR showed that there were no changes in survivin mRNA levels (Fig. 1C). These data suggest that CRP decreases survivin protein level without change of mRNA level in H9c2 cardiac myocytes.

### CRP downregulates Akt/mTOR/p70S6K pathway

**Figure 2:**
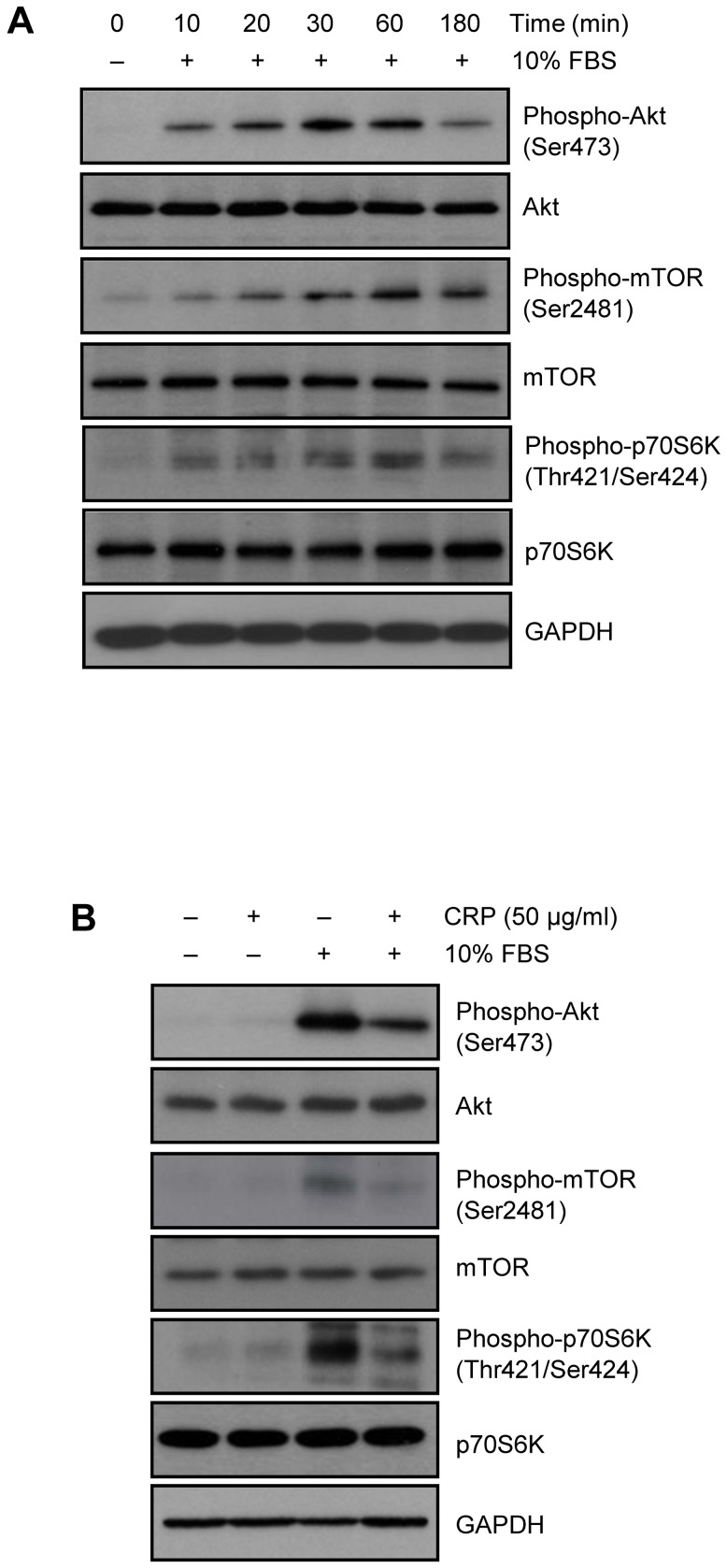
Effect of CRP on the Akt/mTOR/p70S6K pathway in H9c2 cardiac myocytes. (A) After 24 hours of serum starvation, H9c2 cells were treated with 10% FBS for indicated time. Cells were harvested and analyzed for Akt/mTOR/p70S6K signaling pathway by immunoblot assay. (B) H9c2 cells were pretreated for 24 hours with 50 µg/ml of CRP in 0.5% FBS and then treated 10% FBS for 1 hour. The protein levels were analyzed by immunoblot assay.

The regulatory mechanism of survivin expression level through Akt, mTOR and p70S6K signaling has been reported [12], [13]. So we first examined phosphorylation pattern of Akt, mTOR and p70S6K in 10% FBS-treated H9c2 cardiac myocytes. As shown in Fig. 2A, the time-dependent maximum increase in phosphorylation of Akt, mTOR and p70S6K were observed, when incubated with 10% FBS for 1 hour. As shown in Fig. 2B, increased phosphorylation levels of Akt, mTOR, and p70S6K after 10% FBS incubation were significantly reduced, when pretreated 50 µg/ml of CRP for 24 hours. These results suggest that CRP regulates survivin protein level through Akt/mTOR/p70s6k pathway in H9c2 cardiac myocytes.

### PTEN is an upstream target of Akt, mTOR and p70s6k for regulating survivin expression by CRP

**Figure 3:**
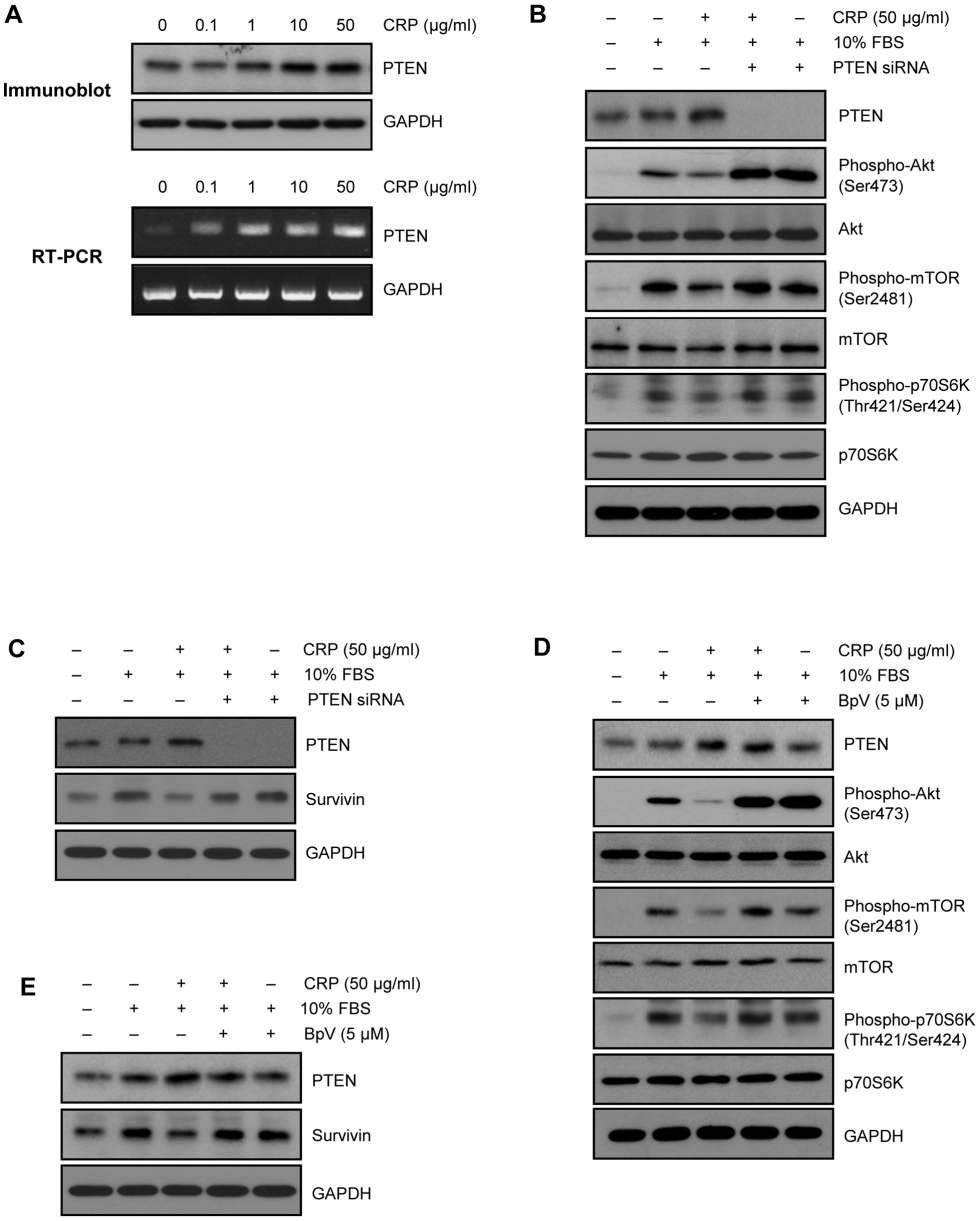
Role of PTEN in CRP-induced downregulation of Akt/mTOR/p70S6K pathway. (A) H9c2 cardiac myocytes were treated with indicated concentration of CRP in 0.5% FBS for 24 hours. Expression levels of PTEN were determined by immunoblot analysis and RT-PCR, respectively. (B) H9c2 cells transfected with 20 nM of PTEN siRNA were incubated 50 µg/ml of CRP in 0.5% FBS for 24 hours and then treated 10% FBS for 1 hour. Cells were harvested and analyzed for PTEN, Akt, mTOR and p70S6K signaling pathway by immunoblot assay. (C) PTEN siRNA-transfected cells were incubated 50 µg/ml of CRP in 0.5% FBS for 24 hours and then treated 10% FBS for 24 hours. Protein levels of PTEN or survivin were analyzed by immunoblot assay. (D) H9c2 cells pretreated with PTEN inhibitor (BpV, 5 µM) for 1 hour were incubated with 50 µg/ml of CRP in 0.5% FBS for 24 hours and then treated 10% FBS for 1 hour. Cells were harvested and analyzed for PTEN, Akt, mTOR and p70S6K signaling pathway by immunoblot assay. (E) PTEN inhibitor-pretreated cells were incubated 50 µg/ml of CRP in 0.5% FBS for 24 hours and then treated 10% FBS for 24 hours. Protein levels of PTEN or survivin were analyzed by immunoblot assay.

Since phosphatase and tensin homologue deleted from chromosome ten (PTEN) regulates PI3K/Akt signal pathway [18], we first examined PTEN level in CRP-treated H9c2 cardiac myocytes. Interestingly, CRP significantly increased protein and mRNA level of PTEN (Fig. 3A). To investigate the role of PTEN in CRP-pretreated H9c2 cardiac myocytes, siRNA for PTEN gene was used to knock-down the expression. Transfection with PTEN siRNA effectively decreased the protein and mRNA level of PTEN in H9c2 cardiac myocytes (Fig. 3B). Downregulation of PTEN with siRNA significantly recovered the decreased phosphorylation of Akt, mTOR, p70S6K, and survivin protein level in CRP-pretreated H9c2 cardiac myocytes (Fig. 3B and C). Moreover, pretreatment with BpV, a specific PTEN inhibitor also recovered the decreased phosphorylation of Akt, mTOR p70S6K, and survivin protein level (Fig. 3D and E). These results indicate that PTEN is an upstream target of Akt/mTOR/p70S6K pathway for regulating survivin protein level.

### CRP increases PTEN expression through activation of p53 by ERK1/2

**Figure 4:**
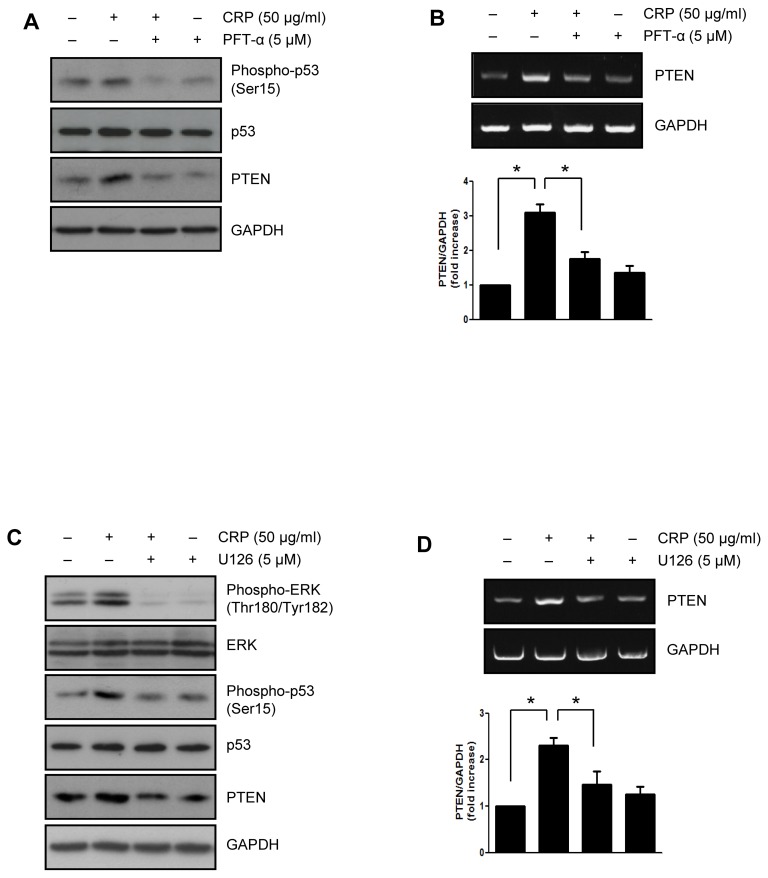
CRP-induced PTEN expression via ERK1/2 and p53 activation. H9c2 cardiac myocytes were pretreated with p53 inhibitor (PFT-α, 5 µM) for 1 hour and then treated with 50 µg/ml of CRP in 0.5% FBS for 24 hours. (A) Expression levels of phspho-p53, p53 and PTEN were analyzed by immunoblot assay. (B) Total RNA was purified from cells and subjected to RT-PCR using primers specific for PTEN. H9c2 cells were pretreated with ERK inhibitor (U0126, 5 µM) for 1 hour and then treated with 50 µg/ml of CRP 0.5% FBS for 24 hours. (C) Expression levels of PTEN were analyzed by immunoblot assay. (D) The level of PTEN was determined by RT-PCR. The results present the means of three independent experiments. Data are mean ± S.E. *P<0.05.

It has been reported that regulation of PTEN transcription by activation of p53 [19]. Recently, we demonstrated that CRP-induced p53 activation is mediated by ERK1/2 which activated through the binding of CRP to FcγRIIIa in H9c2 cardiac myocytes [7]. Therefore, we investigated the effect of several kinase inhibitors on protein and mRNA level of PTEN upregulation induced by CRP treatment. As shown in Fig. 4A and B, when pretreated with p53 inhibitor, PFT-α, the protein and mRNA levels of PTEN were significantly suppressed in CRP-pretreated H9c2 cardiac myocytes. In addition, CRP-induced p53 phosphorylation and PTEN expression were significantly suppressed by treatment with ERK inhibitor, U0126 (Fig. 4C and D). There were no clear changes in PTEN protein level by other inhibitors including ATM/ATR inhibitor, DNA-PK inhibitor, and JNK inhibitor (data not shown).

### CRP inhibits phosphorylation of Akt, mTOR, p70S6K and survivin protein expression through PTEN expression in neonatal rat cardiac myocytes

**Figure 5:**
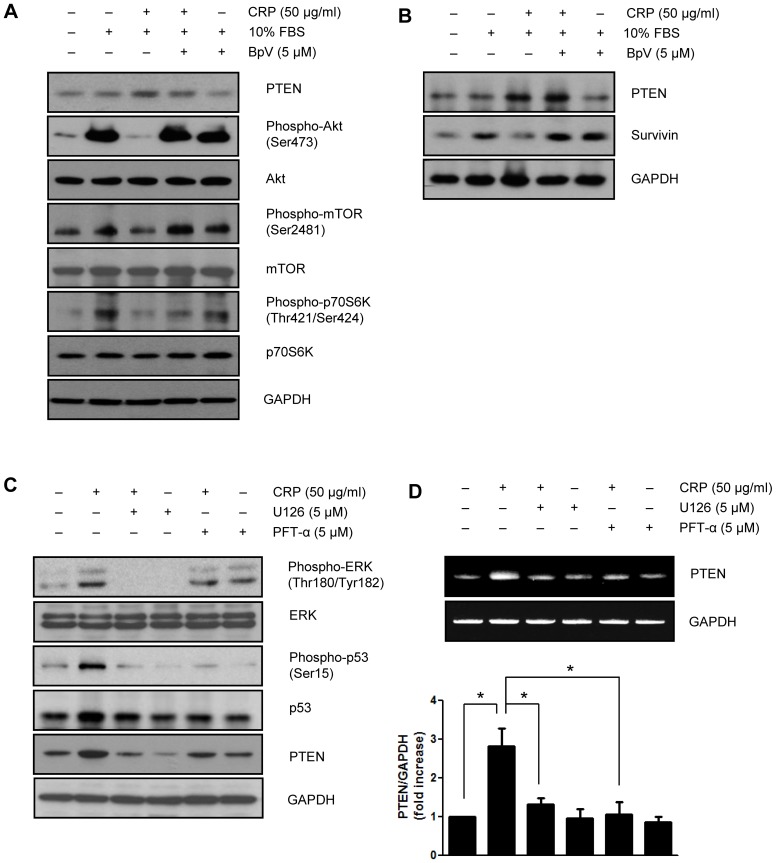
CRP downregulates Akt/mTOR/p70S6K pathway and survivin protein expression through PTEN expression in neonatal rat cardiac myocytes. (A) After 24 hours of serum starvation, neonatal rat cardiac myocytes pretreated with PTEN inhibitor (BpV, 5 µM) for 1 hour were incubated with 50 µg/ml of CRP in 0.5% FBS for 24 hours and then treated 10% FBS for 1 hour. Cells were harvested and analyzed for PTEN, Akt, mTOR and p70S6K signaling pathway by immunoblot assay. (B) PTEN inhibitor-pretreated cells were incubated 50 µg/ml of CRP in 0.5% FBS for 24 hours and then treated 10% FBS for 24 hours. Protein levels of PTEN or survivin were analyzed by immunoblot assay. Neonatal rat cardiac myocytes were pretreated with p53 inhibitor (PFT-α, 5 µM) or ERK inhibitor (U0126, 5 µM) for 1 hour then treated with 50 µg/ml of CRP 0.5% FBS for 24 hours. (C) The protein levels were analyzed by immunoblot assay. (D) Total RNA was purified from cells and subjected to RT-PCR using primers specific for PTEN. The results present the means of three independent experiments. Data are mean ± S.E. *P<0.05.

We also demonstrated the effect of CRP on survivin expression in neonatal rat cardiac myocytes. Moreover, pretreatment with BpV, a specific PTEN inhibitor recovered the decreased phosphorylation of Akt, mTOR p70S6K, and survivin protein level (Fig. 5A and B). In the present study, we have showed that PTEN is an upstream target of Akt/mTOR/p70S6K pathway for regulating survivin protein level in neonatal rat cardiac myocytes. We examined whether PTEN expression was affected by p53 activation in neonatal rat cardiac myocytes. When pretreated with p53 inhibitor, PFT-α, the protein and mRNA levels of PTEN were significantly suppressed in CRP-pretreated neonatal rat cardiac myocytes. In addition, CRP-induced p53 phosphorylation and PTEN expression were significantly suppressed by treatment with ERK inhibitor, U0126 (Fig. 5C and D).

## Discussion

CRP has been considered as an independent predictor of the occurrence and progression of CVD by participating in a variety of inflammatory processes. Furthermore, several reports from several cell types suggest that CRP is a key role in cell differentiation and cell cycle [1], [7], [9], [20]. Survivin, although its mechanism is not clearly understood, is also considered to be a key factor in regulating cell survival and suppression of apoptosis [10]. In our previous study, CRP has no significant apoptotic effect for 24 hours on H9c2 cardiac myocytes [7]. Not only the survivin is selectively expressed at the G2/M phase of the cell cycle in a cell cycle-dependent manner, but also non-cell cycle dependent mechanisms such as signal transducer and activator of transcription 3 or PI3K activity affect survivin expression [8], [13], [21]. PI3K/Akt signaling pathway has been implicated to play an important role in the upregulation of survivin in certain cells [12], [13]. However, there is no information about whether CRP would modulate survivin expression in cardiac myocytes, or which underlying mechanisms are involved. Our study is the first to demonstrate that CRP inhibits survivin expression by PTEN/Akt pathway in cardiac myocytes.

In the present study, we demonstrated that CRP inhibited survivin protein level in a time- and concentration dependent manner, but not survivin mRNA level in cardiac myocytes. Stimulation of cardiac myocytes with CRP for 24 hours induced marked expression of PTEN. Furthermore, knock-down of PTEN using siRNA, or treatment of PTEN inhibitor, restored the decreased survivin protein level induced by CRP. These survivin protein expression levels were correlated with Akt/mTOR/p70S6K activation, suggesting that Akt may be a downstream target of PTEN. Both the ERK1/2 inhibitor and the p53 inhibitor inhibited PTEN expression by CRP. These results may help to understand how CRP affects survivin expression in cardiac myocytes.

The PTEN has been known as a regulator of multiple signal pathways that adjust cell cycle progression, cell proliferation and apoptosis [22], [23]. Also, PTEN is a negative regulator of PI3K/Akt-dependent signaling by dephosphorylating phosphatidylinositol 3,4,5-triphosphate (PIP3) [18], [24], [25]. In the present study, we found that long-term CRP exposure increased endogenous PTEN protein and mRNA level, accompanied by reduced phosphorylation of Akt, mTOR and p70s6k, and reduced survivin protein level in cardiac myocytes. This finding corresponds to the result that chronic exposure to CRP induces PTEN upregulation in endothelial cells [26]. In addition, the decreased protein level of survivin by CRP was considerably reversed by knock-down of PTEN with siRNA or treatment of PTEN inhibitor. These results are in close agreement that PTEN antagonizes the action of PI3K and reduces phosphorylation of downstream signal, Akt, thus leading to the down-regulation of Akt survival signaling pathway [27].

The p53 protein has low levels under normal condition in cells, which exists in a largely inactive state. Activation of p53 in response to various stimuli such as toxin, hypoxia and serum deprivation is associated with an increase in its protein level and phosphorylation activity. In our previous study [7], p53 phosphorylation on Ser15 increased following exposure to CRP in H9c2 cardiac myocytes. We confirmed this finding in the present study, and observed that pretreatment with a specific p53 inhibitor inhibited CRP-induced PTEN expression. The PTEN promoter contains a p53-binding element and PTEN transcription is regulated by activation of p53. And a p53-independent element controlling constitutive expression of PTEN was also identified [19]. ERK1/2 has been shown to act as the upstream kinase for phosphorylation of p53 at Ser15 in response to doxorubicin or CRP in cardiac myocytes or macrophage cells [7], [28]-[30]. In our study, we observed that CRP induces PTEN expression through activation of p53 by ERK1/2 in cardiac myocytes. Pretreatment with an ERK inhibitor resulted in decreased phosphorylation at Ser15 of p53 with decreasing PTEN protein and mRNA level. Therefore, CRP-induced PTEN expression may be regulated by p53 phosphorylation and transactivation through upstream ERK1/2 activation. Our findings support the potential role of CRP as a modulator for survivin expression, and the mechanism by which CRP induces downregulation of Akt/mTOR/p70S6K pathway through expression of PTEN in cardiac myocytes.
